# Limitation of life-sustaining treatment and patient involvement in decision-making: a retrospective study of a Danish COVID-19 patient cohort

**DOI:** 10.1186/s13049-021-00984-1

**Published:** 2021-12-20

**Authors:** Hanne Irene Jensen, Sevim Ozden, Gitte Schultz Kristensen, Mihnaz Azizi, Siri Aas Smedemark, Christian Backer Mogensen

**Affiliations:** 1 Department of Anaesthesiology and Intensive Care, Kolding Hospital, University Hospital of Southern Denmark, Sygehusvej 24, 6000 Kolding, Denmark; 2 Department of Anaesthesiology and Intensive Care, Vejle Hospital, University Hospital of Southern Denmark, Vejle, Denmark; 3 Department of Regional Health Research, University of Southern Denmark, Odense, Denmark; 4 Department of Emergency, Hospital Sønderjylland, University Hospital of Southern Denmark, Aabenraa, Denmark; 5 Emergency Medicine, Hospital of South West Jutland, University Hospital of Southern Denmark, Esbjerg, Denmark; 6 Department of Geriatric Medicine, Kolding Hospital, University Hospital of Southern Denmark, Kolding, Denmark

**Keywords:** End-of-life, Patient involvement, Life-sustaining treatment, COVID-19, Shared decision-making

## Abstract

**Background:**

The coronavirus (COVID-19) pandemic and the risk of an extensive overload of the healthcare systems have elucidated the need to make decisions on the level of life-sustaining treatment for patients requiring hospitalisation. The purpose of the study was to investigate the proportion and characteristics of COVID-19 patients with limitation of life-sustaining treatment decisions and the degree of patient involvement in the decisions.

**Methods:**

A retrospective observational descriptive study was conducted in three Danish regional hospitals, looking at all patients ≥ 18 years of age admitted in 2020 with COVID-19 as the primary diagnosis. Lists of hospitalised patients admitted due to COVID-19 were extracted. The data registration included age, gender, comorbidities, including mental state, body mass index, frailty, recent hospital admissions, COVID-19 life-sustaining treatment, ICU admission, decisions on limitations of life-sustaining treatment before and during current hospitalisation, hospital length of stay, and hospital mortality.

**Results:**

A total of 476 patients were included. For 7% (33/476), a decision about limitation of life-sustaining treatment had been made prior to hospital admission. At the time of admission, one or more limitations of life-sustaining treatment were registered for 16% (75/476) of patients. During the admission, limitation decisions were made for an additional 11 patients, totaling 18% (86/476). For 40% (34/86), the decisions were either made by or discussed with the patient. The decisions not made by patients were made by physicians. For 36% (31/86), no information was disclosed about patient involvement.

**Conclusions:**

Life-sustaining treatment limitation decisions were made for 18% of a COVID-19 patient cohort. Hereof, more than a third of the decisions had been made before hospital admission. Many records lacked information on patient involvement in the decisions.

**Supplementary Information:**

The online version contains supplementary material available at 10.1186/s13049-021-00984-1.

## Background

The coronavirus (COVID-19) pandemic and the risk of an extensive strain of the healthcare systems have made it clear that there is a need to make decisions on the level of life-sustaining treatment for patients requiring hospitalisation, including which patients should be offered care in an intensive care unit (ICU). Patients with advanced illnesses, frailty and old age are more likely to develop severe symptoms of COVID-19 and death [1, 2], and the chance of a successful outcome from ICU therapy may be minimal [3, 4]. If this is the case, transfer to an ICU should be carefully considered for these patients. For patients where ICU therapy is assessed as potentially beneficial, it is important to ensure that this therapy is in accordance with the patient’s wishes regarding life-sustaining treatment [2].

Studies examining end-of-life (EOL) practices have shown that patients’ wishes of the level of life-sustaining treatment are often unknown [5, 6]. A Canadian study surveyed the wishes for the level of life-sustaining treatment of medical patients primarily above 80 years of age, and only 30% were documented in the medical records [7]. Unawareness of patients’ wishes may lead to inappropriate treatment [8–11], but in acute hospitalisation or acute worsening of the patient’s condition during hospitalisation, there may not be the time or option to obtain the patient’s wishes. Therefore, conversations about values and preferences for life-sustaining treatment should occur in a stable period of the illness trajectory whenever possible [12, 13]. As the pandemic has highlighted the need for goal-concordant care [2], it is of interest to examine how EOL practice and limitation decisions are executed in a population of hospitalised patients with COVID-19 to improve our efforts in these critical decisions.

Therefore, the study aimed to investigate the proportion and characteristics of COVID-19 patients with limitation of life-sustaining treatment decisions and the degree of patient involvement in the decisions.

## Methods

### Population and settings

All patients ≥ 18 years of age with COVID-19 as the primary diagnosis admitted to one of three Danish regional university hospitals in 2020. The hospitals have 1296 beds (356, 370, and 570, respectively) and cover most medical specialities. The three hospitals provide all treatments for COVID-19 except Extra Corporeal Membrane Oxygenation (ECMO). None of the hospitals had high-dependency units. At the COVID-19 wards, oxygen treatment was possible as basic treatment with catheter or reservoir mask and as High Flow. One of the hospitals also provided Continuous Positive Airway Pressure (CPAP) treatment at the ward. For all three hospitals, COVID-19 patients in need of non-invasive ventilation (NIV) were transferred to the ICU. The hospitals’ COVID-19 guidelines require that a patient’s level of life-sustaining treatment is assessed upon admission [14].

### Conditions

According to Danish legislation, a patient can decline life-sustaining treatment but cannot demand treatment such as ICU therapy or cardiopulmonary resuscitation (CPR) if this is assessed as futile by the treating physicians. However, if possible, limitation decisions should be discussed with the patient. Furthermore, relatives do not have the legal right to decide on behalf of patients without decision-making capacity. If a patient’s wishes are unknown, the decision regarding the level of life-sustaining treatment is the physicians’ responsibility [15, 16].

### Main outcomes

The primary outcomes were the proportion of patients admitted with COVID-19 and a hospital record documented decision concerning the limitation of life-sustaining treatment before or during admission. Secondary outcomes were the level of patient involvement in the decision-making and characteristics of patients with limitation of life-sustaining treatment decisions.

### Data registration

Lists of all hospitalised patients admitted in 2020 due to COVID-19 were extracted from each hospital’s patient registration system. The registration database included data on age, gender, comorbidities including mental state, body mass index (BMI), frailty assessed by Clinical Frailty Scale [17], number of hospital admissions within the last year, COVID-19 treatment, admission to the ICU, decisions on limitations of life-sustaining treatment (do-not-resuscitate, do not transfer to an intensive care unit, and do-not-intubate) before and during current hospitalisation and how the patients were involved, hospital length of stay, and hospital mortality. The authors developed the database based on literature and former research, and the database was pilot tested on the first ten patients from one of the hospitals to secure that the registration options were exclusive and exhaustive. Questions arising during the data registration, both pilot and later on, were discussed and decided upon within the research group. One researcher from each hospital manually reviewed their admitted COVID-19 patients’ hospital records (all content) and documented the data in the database. Only data from one admission (including transfer to another hospital) was documented in the database. For patients admitted more than once due to COVID-19 in the study timeframe, their most prolonged admission was included. If a patient was transferred to another hospital for specialised COVID treatment, the transfer was included in the registration. Patients who tested positive for COVID-19 during a hospital stay but did not develop any symptoms of COVID-19 or where COVID-19 symptoms had no impact on the illness trajectory were not included in the study.

### Data analyses

All patients with COVID-19 as the main diagnosis admitted to one of the three hospitals in 2020 were included, ensuring a broad COVID-19 population. As the first COVID-19 patients were admitted in March 2020, the actual inclusion time is from March 1st to December 31st 2020. The database was closed 70 days after January 1st 2021. Patients still hospitalised were included with data until the closing of the database. As older patients more often suffer from advanced illnesses and frailty and are the most likely to develop severe symptoms of COVID-19 and death compared to younger patients, sub-analyses were made for patients 70 years or older. Data are presented with descriptive statistics with n (%) for proportions and median/interquartile range (IQR) for ordinal and non-normal distributed continuous variables. Fischer’s exact test and Mann–Whitney U-test were used for comparing patients < 70 years, and patients 70 years or older. The Chi-square test was used to explore associations between patient characteristics and limitation of life-sustaining treatment. All data were analysed in the statistical program Stata 15. A two-sided *p* value < 0.05 was considered significant.

## Results

A total of 476 patients were included in the study, 138 from hospital A, 138 from hospital B, and 200 from hospital C. Of the total number of patients, 24% were admitted in March and April 2020, 3% in May–June, 1% in July–August, 17% in September and October, and 36% in November–December. Hospital length of stay (LOS) was a median of 6 days (interquartile range (IQR) 3–10 days; range 0–101 days). For patients less than 70 years of age, LOS was a median of 5 days (IQR 2–8 days), and for patients 70 years or more, LOS was median 8 days (IQR 5–15 days).

Most of the patients had comorbidities, lived at home and had no home care. Patients 70 years or older were characterised by multiple comorbidities, established home care and often required mobility aids compared with those under 70 years (Table 1). The patient characteristics differed between the three hospitals in gender, age, number of comorbidities, living conditions, frailty and walking function (Additional file 1: Table S1).

**Table 1:** Patient characteristics

	Total n = 476^a^	Age < 70 n = 258^a^	Age 70 + n = 218^a^	*p* values^b^
Gender. Female n (%)	208 (44)	124 (48)	84 (39)	0.04
Age. Median (IQR)	68 (57–79)	55 (45–61)	79 (74–83)	
BMI. Median (IQR)	28 (24–32)	30 (25–33)	26 (23–30)	< 0.001
Comorbidities^c^ n (%)				
None	102 (21)	94 (36)	8 (4)	
Diabetes	89 (19)	41 (16)	48 (22)	
Heart diseases^d^	359 (75)	94 (36)	265 (122)	
Lung diseases^e^	102 (21)	42 (16)	60 (28)	
Cancer	43 (9)	15 (6)	28 (13)	
Dementia	20 (4)	0 (0)	20 (9)	
Other	314 (66)	139 (54)	182 (83)	
Number of comorbidities. Median (IQR)	2 (1–3)	1 (0–2)	3 (1–4)	< 0.001
Number of admissions within last year Median (IQR)	0 (0–1)	0 (0–0)	0 (0–1)	< 0.001
Living conditions. n (%)				< 0.001
Lives at home alone	119 (25)	51 (20)	68 (31)	
Lives at home with others	304 (64)	187 (72)	117 (54)	
Lives in care facility	22 (5)	1 (0.5)	21 (10)	
Other	18 (4)	9 (3)	9 (4)	
Home care^c^. n (%)				
None	365 (77)	239 (93)	126 (58)	
Help with cleaning	60 (13)	10 (4)	50 (23)	
Help with medicine	61 (13)	11 (4)	50 (23)	
Help with personal hygiene	51 (11)	8 (3)	43 (20)	
Help with food	32 (7)	4 (2)	28 (13)	
Other	35 (7)	5 (2)	30 (14)	
Clinical frailty scale^f^. Median (IQR)	2 (2–4)	2 (1–3)	3 (2–5)	< 0.001
Walking function. n (%)				
Walks without aids	371 (78)	240 (93)	131 (60)	< 0.001
Walks with walking stick	14 (3)	4 (2)	10 (5)	
Walks with zimmer frame	56 (12)	6 (2)	50 (23)	
Other walking aids	12 (3)	2 (1)	10 (4)	
No walking function	10 (2)	1 (0.5)	9 (4)	

^a^Different n for individual variables due to missing data

^b^Fischer’s exact test for categorical data and Mann–Whitney U-test for ordinal and continuous, non-normal distributed data

^c^Possible to choose more than one answer

^d^Hypertension, Ischemic heart disease, heart failure, AFLI. Percentage may be > 100 due to some patients having more than one heart disease

^e^Chronic obstructive pulmonary disease, Asthma

^f^Clinical frailty scale: 1:Very fit; 2:Well; 3:Managing well; 4:Vulnerable; 5:Mildly Frail; 6: Moderately frail; 7:Severly frail; 8:Very severely frail; 9:Terminally ill

The majority of patients received oxygen therapy and were discharged to home (Additional file 1: Table S2). The hospital mortality rate was 4% (19/476); 2% (5/258) for patients < 70 years of age, and 6% (14/218) for patients aged 70 years or older. Additionally, eight patients (2%) had life-sustaining treatment withdrawn and were discharged to be allowed to die at home.

From March 2020 to February 2021, 263 nursing home residents in the hospitals’ region were infected with COVID-19, of which 73 died of the infection [18]. The three hospitals in the present study cover 57% of all nursing home residents in the region [19]. Our study included 21 nursing home residents admitted to hospital due to infection with COVID-19. This indicates that some COVID-19 infected nursing home residents were not hospitalised.

### Limitation of life-sustaining treatment

**Fig. 1:**
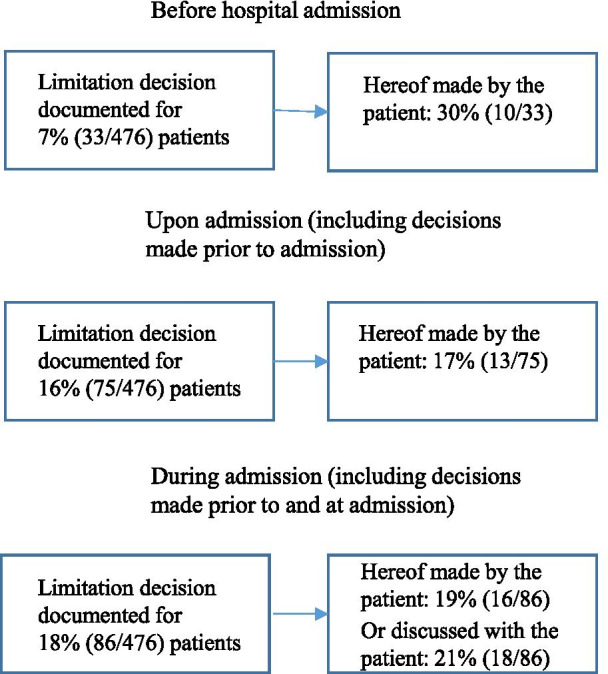
Documentation of limitation of life-sustaining treatment decisions and patient involvement herein

As shown in Fig. 1 decisions about limitation of life-sustaining treatment were made prior to admission, at admission, and during the admission. A decision was made for 18% of patients, the most common being limitation to the ward. In 19% hereof, it was documented that the patient made the decision, and for additionally 21%, that the decision was discussed with the patients. For 36%, no information was disclosed about patient involvement. Almost all of the limitation decisions were made for patients aged 70 years or older (Table 2).

**Table 2:** Level of life-sustaining treatment during hospital admission

	Total		Age < 70		Age 70 +	
	n = 476	%	n = 258	%	n = 218	%
Level of life-sustaining treatment						
Full treatment^a^	390	(88)	252	(98)	138	(63)
ICU with MV but no CPR	8	(2)	1	(0.5)	7	(3)
No escalation to the ICU^b^	60	(13)	4	(2)	56	(26)
Palliation only	18	(4)	1	(0.5)	17	(8)

	Total		Age < 70		Age 70 +	
	n = 86	%	n = 6	%	n = 80	%
*Patients with limitation*						
Decision-maker						
Physician	70	(81)	5	(83)	65	(81)
Patient	16	(19)	1	(17)	15	(19)
Decision discussed with patient						
Yes, patient made decision	16	(19)	1	(17)	15	(19)
Yes	18	(21)	3	(50)	15	(19)
Not possible	21	(24)	1	(17)	20	(25)
Not disclosed	31	(36)	1	(17)	30	(38)

*ICU* intensive care unit, *MV* mechanical ventilation, *CPR* cardiopulmonary resuscitation

^a^Includes both patients where “Full treatment” is ordered in the hospital record and those where no limitations are described or initiated

^b^Including no CPR

For 43% (203/476) of hospital records, no specific information about the level of life-sustaining treatment was provided at admission: in 53% (137/258) for patients < 70 years, and 30% (66/218) for patients 70 years or older).

For many of the patients, full treatment was an implicit order, as it was not documented as such in the hospital records, but was based on the treatment offered. For the whole “Full treatment group” (n = 390), 3% of the hospital records included registration of discussion with the patient about the level of life-sustaining treatment.

The percentage of patients with limitation decisions decreased from March–August (41/131; 31%) to September–December (45/345; 13%) (*p* < 0.001). For patients with limitation decisions, a significantly larger number of patients admitted from September to December (12/45; 27%) made the decision compared to patients admitted from March–August (4/41; 10%) (*p* = 0.044).

In the whole period, no triage due to lack of ICU beds was needed.

### Factors associated with limitations

Age, number of comorbidities, level of frailty, need for walking aids and living conditions were the main variables associated with decisions of treatment limitations (Table 3).

**Table 3:** Factors associated with one or more limitations of treatment

	Limitations. Yes		Limitations. No		*p* value^c^
	n = 86^a^	%	n = 363^a,b^	%	
Gender					
Female	31	(36)	162	(45)	0.15
Male	55	(64)	201	(55)	
Age					
< 70 years	6	(7)	232	(64)	< 0.001
70+ years	80	(93)	131	(36)	
BMI^d^					
< 18.5	5	(7)	3	(1)	0.007
18.5–25	30	(43)	75	(32)	
> 25–30	19	(28)	73	(31)	
> 30	15	(22)	81	(34)	
Comorbidities					
None	0	(0)	58	(25)	< 0.001
One comorbidity	7	(14)	63	(27)	
Two comorbidities	17	(33)	78	(33)	
> Two comorbidities	27	(53)	37	(16)	
Clinical frailty scale^e^					
1–2	3	(4)	285	(90)	< 0.001
3–5	49	(65)	27	(9)	
6–9	24	(32)	4	(1)	
Walking function					
No aids	17	(20)	332	(94)	< 0.001
Walk with aid	58	(69)	19	(5)	
No walking function	9	(11)	1	(0.5)	
Living conditions					
Live at home^f^	59	(69)	339	(97)	< 0.001
Live in care facilities	18	(21)	3	(1)	
Other	8	(9)	9	(3)	

^a^Different n in individual variables due to missing data

^b^Twenty-seven of 390 patients were excluded from the analysis due to lack of disclosure of the level of life-sustaining treatment

^c^Chi-square test

^d^Body mass index

^e^Clinical frailty scale: 1:Very fit; 2:Well; 3:Managing well; 4:Vulnerable; 5:Mildly Frail; 6: Moderately frail; 7:Severly frail; 8:Very severely frail; 9:Terminally ill

^f^Alone or with someone

## Discussion

In these Danish COVID-19 patients, one or more limitations of treatment were documented for 18% of the patients, and of those, 19% of the decisions were documented as made by the patient. In the group of patients where limitation of life-sustaining treatment were documented, less than half of the cases were discussed with the patient. A high number of the patient records lacked specific instructions about the level of life-sustaining treatment, despite the fact that all hospitals’ COVID-19 guidelines required that the level of life-sustaining treatment was assessed upon admission.

The high number of hospital records where the level of life-sustaining treatment was not specified could cause uncertainty in a medical crisis. Danish legislation states that if a do-not-resuscitate order has not been documented, CPR must be initiated [15, 16]. If the level of life-sustaining treatment has not been assessed and decided upon ahead of time, this may lead to inappropriate care [10, 20] and moral distress for healthcare professionals [21]. Conversations about life-sustaining treatments can lead to less aggressive EOL care [12, 13, 22], reduce health care costs [23], and may even lead to more prolonged survival [24]. Studies have shown that many patients have thought about life-sustaining treatment and EOL care, but less than a third have discussed their preferences with physicians [7, 25]. Physicians may be afraid of distressing patients by bringing up the issue of life-sustaining treatment and death [26]. However, studies have shown no differences in the level of fear and anxiety in a group of terminally ill cancer patients, who discussed EOL, compared to a group that did not discuss EOL matters [27], and families were more uncomfortable than the patient talking about advanced care planning [28]. Finding the most appropriate time to conduct a conversation about patients’ values and preferences for life-sustaining treatment may be challenging. Even though these conversations need not be lengthy [29], an acute care setting is often not ideal for either the patient or the healthcare professional [30]. In the current study, almost a third of the treatment limitation decisions upon admission were made before the admission. Conducting the conversation in a stable period in the patient’s illness trajectory with a physician who knows the patients is preferred [26, 31]. Another issue is identifying patients with whom a conversation about wishes for life-sustaining treatment is pertinent [32, 33]. The “surprise question” (would I be surprised if this patient died in the next 12 months) is a poor to modest predictive tool for death [34], but may still be useful for timing a conversation for levels of treatment. A more specific tool is the Supportive and Palliative Care Indicators Tool (SPICT) which is a practical, clinical tool to help healthcare professionals identify patients at risk of deteriorating and dying [35].

Different models to clarify patients’ wishes and preferences exist such as the British Recommended Summary Plan for Emergency Care and Treatment (ReSPECT) [36], the American Physician Orders for Life Sustaining Treatment (POLST) [37], and a variety of other advanced care planning tools [38]. Clarifying patients’ wishes is possible also in an epidemic. In a retrospective analysis of routinely collected data from ReSPECT records in 2019 compared to April 2020, Hurlow et al. found an increased use of the ReSPECT form during the first part of the pandemic with a greater proportion of plans discussed with the patient compared to 2019 [39].

Although Danish family members do not have legal rights to make decisions on behalf of the patient without decision-making capacity, family members are involved as the patient’s advocate in presenting the patient’s values and preferences. However, several studies have shown discrepancies between patients’ and families opinions [40], and therefore discussions with old, sick and frail patients about values, wishes, and preferences should be conducted in a stable disease period whenever possible.

The number of hospital records in the current study lacking information about patient involvement in a limitation decision may indicate that some decisions have been made without involving the patients. ICU therapy and CPR should only be offered to patients with a realistic hope of success, which is an assessment and decision made by physicians in Denmark, but this should be discussed with the patient. In the current study, limitation decisions were made for 37% of the patients 70 or more years of age. The whole group of patients aged 70 years or older had a median of three comorbidities and a median clinical frailty score of three. It would have been relevant to have had conversations about values and preferences with more patients from this group, as research has shown discordance between physicians’ assessment of patient goal of care with the patients’ own goals of care [41]. The current study aimed to explore the levels of limitation decisions and shared decision-making, but in future studies, it will be relevant to include patient involvement in decisions about full treatment, including transfer to an ICU.

The decrease in percentage of limitation decisions from the first part of the registration period compared to the last part of the period may partly be due to improved treatment options and experiences of treating COVID-19 patients [42]. The authors assess that limitation decisions for COVID-19 patients were made on identical foundation as other patients, and that the COVID-19 patients were offered treatment to similar extents as patients admitted with severe pneumonia.

Patient involvement, including shared decision-making, has become a key topic in healthcare, and this also includes patient involvement (if possible) in decisions regarding the level of life-sustaining treatment. However, this warrants an individual approach to ensure that the level of involvement is in accordance with the patient’s wishes [43, 44]. The results from this study present current practice and will hopefully promote further interest in patient involvement in shared decision-making about the level of life-sustaining treatment.

Strengths of the study include data from three hospitals and an entire 2020 cohort. Limitations include the retrospective access to hospital record data, which may result in information bias, especially in regard to lack of information about circumstances for decision-making and lack of documentation of patient involvement. Comorbidities were not weighted according to severity, and the Clinical Frailty Score was based solely on hospital record data without a clinical assessment. Likewise, the retrospective design prevents the possibility of assessing whether discussing a limitation decision with a patient was a possibility. There is probably collinearity in the factors found to be associated with limitations of treatment. However, due to the fairly limited number of patients with limitations included in the study, only unilineal analyses were performed. Apart from data on COVID-19 related nursing home deaths, no information was available on COVID-19 positive patients not admitted to the hospitals. Although a pilot registration was conducted and interpretation issues were discussed continuously in the research group, some subjectivity may exist in the manual review method. The study was conducted in Denmark where the legal frame for decision-making is different from for example the United States in regard to family involvement in decision-making. However, the need for timely conversations with patients with decision-making capacity regarding values and preferences and documentation hereof are relevant for all health professionals around the world.

## Conclusion

Treatment limitation decisions were made for approximately a fifth of the COVID-19 patients. Of these patients, more than a third of the decisions had been made before hospital admission. Approximately half of the patients either made the treatment limitation decision themselves or were involved in the decision. However, many hospital records lacked information about level of life-sustaining treatment and patient involvement. The study underlines the need for a pro-active approach to clarification of patients’ values, goals, and preferences for level of life-sustaining treatment and the need for a more extensive documentation of decision-making.

## Supplementary Information

**Additional file 1.** **Table S1**. Patient characteristics divided between hospitals and **Table S2**. Treatment and discharge data.

## Data Availability

The dataset used and analysed during the current study are available from the corresponding author on reasonable request.
