# Supplementary material for: Limitation of life-sustaining treatment and patient involvement in decision-making: a retrospective study of a Danish COVID-19 patient cohort
Source: Scand J Trauma Resusc Emerg Med. 2021 Dec 20;29:173. doi: 10.1186/s13049-021-00984-1 (PMC8686092; doi:10.1186/s13049-021-00984-1)
Supplement: Supplementary file 1 — Additional file 1. Table S1. Patient characteristics divided between hospitals and Table S2. Treatment and discharge data. [file 13049_2021_984_MOESM1_ESM.docx]

**Limitation of treatment and patient involvement in decision-making: a retrospective descriptive study of Danish COVID-19 patients**

**Appendix 1. Patient characteristics divided between hospitals**

**Appendix 2. COVID-19 treatment**

**Appendix 1. Patient characteristics divided between hospitals**

**Table S1. Patient characteristics for each participating hospital**

|  | Hospital A | | Hospital B | | Hospital C | |  |
| --- | --- | --- | --- | --- | --- | --- | --- |
|  | n=138^1^ | | n=138^1^ | | n=200^1^ | | p-value^2^ |
| Gender. Female n(%) | 58 | (42) | 74 | (54) | 76 | (38) | 0.02 |
| Age. Median (IQR) | 70 | (57-80) | 62 | (47-75) | 68 | (57-79) | 0.004 |
| BMI. Median (IQR) | 28 | (24-32) | 29 | (24-33) | 27 | (24-30) | 0.34 |
| Comorbidities^3^. n(%) |  |  |  |  |  |  |  |
| None | 23 | (17) | 32 | (23) | 47 | (24) |  |
| Diabetes | 22 | (16) | 26 | (19) | 41 | (21) |  |
| Heart diseases^4^ | 116 | (84) | 81 | (59) | 162 | (81) |  |
| Lung diseases^5^ | 33 | (24) | 17 | (12) | 46 | (23) |  |
| Cancer | 11 | (8) | 14 | (10) | 18 | (9) |  |
| Dementia | 3 | (2) | 7 | (5) | 10 | (5) |  |
| Other | 89 | (64) | 60 | (43) | 135 | (68) |  |
| Number of comorbidities. Median (IQR) | 2 | (1-4) | 1 | (1-3) | 1 | (0-3) | <0.001 |
| Number of admissions within last year. Median (IQR) | 0 | (0-1) | 0 | (0-1) | 0 | (0-1) | 0.17 |
| Living conditions. n(%) |  |  |  |  |  |  | 0.01 |
| Lives at home alone | 45 | (34) | 36 | (27) | 38 | (19) |  |
| Lives at home with others | 73 | (55) | 92 | (69) | 139 | (71) |  |
| Lives in care facility | 7 | (5) | 4 | (3) | 11 | (6) |  |
| Other | 8 | (6) | 1 | (1) | 9 | (5) |  |
| Home care^3^. n(%) |  |  |  |  |  |  |  |
| None | 99 | (72) | 113 | (82) | 153 | (77) |  |
| Help with cleaning | 33 | (24) | 8 | (6) | 19 | (10) |  |
| Help with medicine | 26 | (19) | 17 | (12) | 18 | (9) |  |
| Help with personal hygiene | 21 | (15) | 12 | (9) | 18 | (9) |  |
| Help with food | 20 | (14) | 4 | (3) | 8 | (4) |  |
| Other | 5 | (4) | 17 | (12) | 13 | (7) |  |
| Clinical Frailty Scale^6^. Median (IQR) | 3 | (2-5) | 2 | (1-3) | 2 | (2-4) | <0.001 |
| Walking function. n(%) |  |  |  |  |  |  | 0.03 |
| Walks without aids | 101 | (77) | 122 | (90) | 148 | (76) |  |
| Walks with walking stick | 3 | (2) | 3 | (2) | 8 | (4) |  |
| Walks with zimmer frame | 18 | (14) | 8 | (6) | 30 | (15) |  |
| Other walking aids | 7 | (5) | 0 | (0) | 5 | (3) |  |
| No walking function | 3 | (2) | 3 | (2) | 4 | (2) |  |

1. Different n for individual variables due to missing data

2. Chi square test for gender, living condition and walking function, otherwise Kruskall-Wallis.

3. Possible to choose more than one answer

4. Hypertension, Ischemic heart disease, heart failure, AFLI. Percentage may be > 100 due to some patients having more than one

heart disease.

5. Chronic obstructive pulmonary disease, Asthma

6. Clinical Frailty Scale: 1:Very fit; 2:Well; 3:Managing well; 4:Vulnerable; 5:Mildly Frail; 6: Moderately frail; 7:Severly frail;

8:Very severely frail; 9:Terminally ill.

Hospital lengths of stay was a median of seven days (interquartile range 3-12) for Hospital A, five days (IQR 2-9) for hospital B, and six days (IQR 3-10) for Hospital C.

**Appendix 2. COVID-19 treatment**

**Table S2. Treatment and discharge data**

|  | Total | | Age < 70 | | Age 70 + | |
| --- | --- | --- | --- | --- | --- | --- |
|  | n=476 | % | n=258 | % | n=218 | % |
| Oxygen |  |  |  |  |  |  |
| None | 158 | (33) | 103 | (40) | 55 | (25) |
| In ward | 188 | (40) | 101 | (39) | 87 | (40) |
| Hudson/Highflow/NIV/CPAP^1^ in ward | 64 | (14) | 19 | (7) | 45 | (21) |
| NIV/CPAP ICU | 5 | (1) | 3 | (1) | 2 | (1) |
| MV^2^ ICU | 61 | (13) | 32 | (12) | 29 | (13) |
| Remdicivir (Yes) | 186 | (39) | 109 | (39) | 85 | (39) |
| Steroid (Yes) | 217 | (46) | 114 | (44) | 103 | (47) |
| Discharge status |  |  |  |  |  |  |
| Home | 397 | (83) | 243 | (94) | 154 | (71) |
| Temporary care facilities | 14 | (3) | 2 | (1) | 12 | (6) |
| Other | 46 | (10) | 8 | (3) | 38 | (17) |
| Death in hospital | 19 | (4) | 5 | (2) | 14 | (6) |

1. NIV=Non-invasive ventilation; CPAP=Continuous positive airway pressure; ICU=Intensive Care Unit

2. MV= Mechanical ventilation
